# Do Drugs Used in Obstetric Anesthesia Interfere with Early Breastfeeding? What Determines Drug Safety in Lactation? Part 1

**DOI:** 10.34763/devperiodmed.20192304.227232

**Published:** 2021-01-29

**Authors:** Maria Wilińska, Wojciech Walas, Ewa Kamińska, Małgorzata Malec Milewska, Agnieszka Sękowska, Ewa Helwich

**Affiliations:** 1 Department for Neonatology, Prof. W. Orłowski Independent Public Clinical Hospital, Centre of Postgraduate Medical Education, Warsaw, Poland; 2 Department for Anesthesiology and Intensive Care Unit for Children and Neonates, Clinical University Hospital, Opole, Poland; 3 Department of Pharmacology, Institute of Mother and Child, Warsaw, Poland; 4 Department for Anesthesiology and Intensive Care, Prof. W Orlowski Independent Public Clinical Hospital, Centre of Postgraduate Medical Education, Warsaw, Poland; 5 Department for Neonatology and Neonatal Intensive Care, Institute of Mother and Child, Warsaw, Poland

**Keywords:** anesthesia, delivery, cesarean section, drugs, breastfeeding, znieczulenie, poród, cięcie cesarskie, leki, karmienie piersią

## Abstract

The issues concerning the transfer of drugs into mothers' milk and their influence on breastfed babies have not been fully studied. Assessment of the situation should include such aspects as drug transfer into fetal blood and into mother's milk, the real risk of inhibiting lactogenesis 2 in women after birth, as welI as the psychological consequences for the mother of suspending breastfeeding. The risk of feeding a newborn with formula based on cow's milk is another fateful issue.

The following paper presents the pharmacokinetic characteristics of drugs which determine their transfer level through the placenta and into mother's milk during the perinatal period and lactation.

## Streszczenie

Zagadnienia związane z przenikaniem leków do pokarmu matki oraz ich wpływem na karmione piersią dziecko nie są w pełni poznane. Realna ocena sytuacji powinna uwzględniać kilka aspektów, jak transfer leków do krwi płodu oraz do mleka matki, realne ryzyko zahamowania /aktogenezy li u kobiety po porodzie, a także skutki psychiczne dla matki związane ze wstrzymaniem przystawiania dziecka do piersi. Problemem osobnym, potencjalnie brzemiennym w skutki, jest narażenie noworodka na ryzyko karmienia mieszanką sztuczną opartą na mleku krowim.

W niniejszym artykule autorzy przedstawiają charakterystykę właściwości farmakokinetycznych leków, które decydują o stopniu przenikania leku przez łożysko i do mleka matki w okresie okołoporodowym i podczas laktacji.

## Introduction

Starting successful lactation demands regular breastfeeding of newborns immediately after birth. Systematic emptying of the breasts since the first hours following labor is essential for stimulating effective lactation [1]. The highest percentage of breastfeeding was noticed after spontaneous labors, especially in home births. Latch-on after cesarean section happens later and formula feeding is used more often [2].

According to WHO, the optimal percentage of cesarean sections should account for 10-15% deliveries [3], whereas *The Lancet* reports that the number of cesarean sections in the world doubled between 2000 and 2015 [4] and accounts for 21.1% of all live births. The fastest growth of cesarean sections is observed in Eastern Europe, Central Asia (annual growth average of 5.5%) and Southern Asia (6.1%).

In Poland, the Statistical Yearbook does not provide information concerning cesarean sections. The data gathered in 2016 by the Foundation for Childbirth with Dignity shows that the percentage of labors ended with cesarean section is very high: it accounts for 45.8% of the total number of births and is described as disastrous. It is one of the highest rates in Europe [5].

## Anesthetic procedures in obstetrics

Anesthesia - the induction of a medical condition that makes it possible to perform surgery or other potentially painful and unpleasant medical procedures by interrupting the conduction of afferent nerve impulses from the receptor and efferent cells to effector cells. Depending on the method, we differentiate between general and regional anesthesia and there is also a combined version, which joins both. General anesthesia, or narcosis, is composed of various phases which are induced by different substances. It involves:

- loss of consciousness (hypnosis) and provoking amnesia,
- elimination of pain (analgesia),
- loss or weakening of physiological, somatic, somato-vi-sceral and autonomic response to stimuli (areflexia),
- muscle relaxation (relaxatio).

Regional anesthesia is a temporary (reversible) blockage of nerves or other nervous structures in the area of the planned surgery. This method makes it possible to perform surgery while retaining the patients consciousness. It is, however, usually used together with sedative drugs. Combined anesthesia joins the techniques of general and regional anesthesia in order to increase the effectiveness of reducing adverse effects.

Sedation puts the patient in a state of calm, reduces anxiety and excitement without losing her cooperation and defense reflexes.

Analgosedation - sedation with additional pharmacological pain relief.

## Analgesia during labor

As far as the safety of the fetus, newborn and natural feeding is concerned, analgetic drug administration to mothers in the course of spontaneous labor, cesarean section or removal of the rest of the placenta is essential.

Anesthetic procedures must consider the differences between various medical procedures and the influence of anesthetics on the contractile activity of the uterus. Drugs used before umbilical cord clamping may influence the newborns condition. Drugs administered before and after cutting the umbilical cord impact natural breastfeeding.

## Spontaneous labor analgesia

Nowadays it is extremely rare to use drugs with a general effect in order to numb or relieve pain during labor. Regional anesthesia is used instead. Continuous epidural anesthesia is claimed as the gold standard [6, 7]. Adjusting the proper concentration and dose of regional anesthesia and opioids makes it possible to manage the strength and range of anesthesia. A spinal block is used less often.

Moreover, obstetricians less frequently use paracervical block and prudential block. There are a few reports that describe non-pharmacological methods of relieving pain, such as transcutaneous electrical nerve stimulation (TENS), massage or acupuncture [8].

## Cesarean section analgesia

Cesarean section analgesia is determined by various factors concerning the safety of the woman in labor, the fetus and the newborn. Planned cesarean section may be performed under general or regional anesthesia, epidural or spinal anesthesia. Cesarean section due to sudden medical indications is usually performed under general anesthesia.

## Analgesia for removing the rest of the placenta from the uterus

This particular anesthesia has no direct influence on the child, but is essential as far as lactation is concerned.

## Breastfeeding and analgesia

Drugs used in obstetrics raise fears both in medical personnel and parents. Drug administration in women during spontaneous labor, anesthesia during cesarean section and while removing the rest of the placenta from the uterus may be the reason for delaying the first breastfeeding. The literature review shows that the concerns of medical personnel are exaggerated and result from an over-cautious approach towards breastfeeding. Although almost all drugs excrete to human milk, adverse effects in newborns are rare and mild.

According to the Summary of Product Characteristics (SmPC), the use of 90% of all drugs should be limited both during pregnancy and breastfeeding. There is no information legitimizing such recommendations [9].

The risks of drug use in the woman giving birth connected with the transfer of medications into milk and their influence on lactation may not be considered without understanding the benefits of breastfeeding for both the mother and the child. Colostrum is a product of lactogenesis 1 which is characteristic for the third trimester of pregnancy. Its composition is unique and will never be repeated during any stage of lactation. The health-promoting properties of colostrum are very high, especially in terms of a high concentration of lactoferrin, lysozyme, sIgA and oligosaccharides, and an extremely high antioxidant potential. At every stage of lactation breast milk has an optimal nutritious composition that adapts to the current needs of the baby. Due to its bioactive components, it stimulates the growth and maturity of various tissues and organs. It also presents anti-inflammatory, immunological and immunostimulating effects. As a result, mother’s milk ensures proper metabolic programming, it reduces the risk of cardiovascular, gastrointestinal, endocrine diseases and cancers. It also supports proper psychomotor development. The positive effect on women’s organisms includes many aspects concerning faster postpartum stabilization. Long-term benefits include the reduction of some cancer risks in women and an increase in bone mineral density at the perimenopause period. Delaying the beginning of breastfeeding interferes with initiating lactogenesis 2, which is responsible for milk production after birth [7, 8, 9].

While studying drug administration in mothers at lactation, it is necessary to consider not only missing the benefits provided by breastfeeding, but also the risk of using formula based on cow’s milk. Using the protein of another species as the first food in a baby’s diet and the continuation of formula feeding leads to an increased risk of allergic diseases. It also causes negative biological effects that result in well-known consequences of non-physiological metabolic programming [10].

The following paper analyzes the pharmacokinetic properties of drugs administered to women giving birth, while using spontaneous labor and cesarean section analgesia and the potential impact of the drugs that have been administered on the newborns’ clinical condition at birth and during breastfeeding.

## Factors affecting drug concentration in mother's milk and fetal blood [11, 12, 13, 14, 15]

Clinicians need to learn the factors which modify the mechanisms of drug transfer into fetal blood and mother’s milk.

1. Drug concentration in the mothers blood Drug concentration in the mother’s blood is the most important factor determining the level of transfer into milk. There is constant, bilateral transport that tries to rebalance drug concentrations in both blood and milk. Passive diffusion is a basic mechanism of transferring the drug from the mother’s blood into milk and through the placenta into fetal circulation. Apart from passive diffusion in the water phase, drug lipophilicity facilitates drug transport through phospholipid barriers and to mother’s milk lipids.
  Drug concentration in the mother’s blood is modified by its pharmacokinetics, especially by its distribution to various tissues and organs. A high volume of distribution is connected with highly decreasing drug concentration in the mother’s blood and also in the mother’s milk.
  Drug transfer into milk depends on the extent of the mother’s plasma protein binding. Only the free drug fraction can diffuse through biological barriers. It easily reaches high concentration in various body fluids. The fraction which is highly bound to plasma proteins (e.g. sertraline) stays in circulation and does not reach significant concentration in tissues and other fluids.
2. The particle size of the drug Small particle substances, such as alcohol, nicotine or caffeine represent low-molecular-weight compounds that easily combat biological bariers. They reach high concentration in fetal blood and in mother’s milk. Drugs with high molecular weight (insulin, heparin) mostly stay in the original compartment.
3. Ionization level
  Only a non-ionized drug form combats biological membranes . Milk is characterized by a more acid reaction (pH approx. 7.2) than mother’s blood (pH approx. 7.4). This situation enables drugs, such as oxycodone or codeine, to transfer into milk. These drugs get into the so-called ion trap and accumulate in mother’s milk. Conversely, organic acids, such as penicillin, are ionized and they show the tendency to persist in the mother’s circulation.
4. Mother’s pharmacogenomics

The influence of mother’s pharmacogenomics may be observed in the metabolism of codeine. During this process codeine is transformed by p450 cytochrome to morphine. An ultra-rapid metabolism phenotype is present in 10% of the women from Eastern Europe and 30% of the women in Africa. In these women, repeated codeine doses are connected with high endogenous morphine production. Morphine transport into mother’s milk is easy and fast. A high drug concentration may cause depression of the central nervous system in the fetus and in the breastfed baby. Breastfeeding women should not take codeine, even in the form of a fixed combination. The alternative analgesia, with a much safer profile, is paracetamol and ibuprofen. These drugs used in monotherapy are effective only in treating mild to moderate pain.

**Table I:** The factors affecting drug concentration in mother's milk and in the child's blood. Tabela I. Czynniki wpływające na stężenie leków w mleku matki i krwi dziecka.

Drug dependent factors	Mother dependent factors	Child dependent factors
*Czynniki zależne od leku*	*Czynniki zależne od matki*	*Czynniki zależne od dziecka*
Dose		
*Dawka*		
	Drug concentration in mother's blood	Maturity of the organism
Lipophilicity	*Stężenie leku we krwi matki* - route of administration	*Dojrzałość organizmu*
*Lipofilność*	*droga podania*	Preterm newborns
	- ability to metabolize the drug	*Noworodki urodzone przedwcześnie*
	*zdolność do metabolizowania leku*	- lower activity of liver enzymes
Molecular weight	- binding proteins concentration	*mniejsza aktywność enzymów*
*Masa cząsteczkowa*	*stężenie białek ważących*	*wątrobowych*
Ability to bind to proteins		- decreased glomerular filtration *zmniejszona filtracja kłębkowa*
*Zdolność wiązania z białkami*		- slower urinary excretion
Ability to ionize	Genetic factors	*wolniejsze wydalanie nerkowe*
*Zdolność do jonizacji*	*Czynniki genetyczne*	
Half-life	Mammary gland construction	
*Okres półtrwania*	*Budowa gruczołu piersiowego*	
Bioavailability	Progesterone concentration	Binding protein concentration
*Biodostępność*	*Stężenie progesteronu*	*Stężenie białek więżących*

Table I presents the summary of the factors determining the transfer level of drugs from mother’s blood into her milk and factors influencing the drug level in the child’s blood.

## The pharmacokinetics of drugs in breastfeeding women

- RID (Relative Infant Dose).

Drug intake in a breastfed child while feeding is the multiplication of drug concentration in mother’s milk (C _)_ and the milk volume which the baby consumed in a day (V_m_). In order to objectify milk volume, one may calculate it per kilogram of the baby’s body weight. For instance, if the drug concentration in milk amounts to 50 mcg/L and the baby consumes 0.15 L of milk per kilogram, it means that the baby gets 7.5 mcg/ kg/day of the drug. Five feedings a day mean 1.5 mcg/kg of drug per feeding.

The relative drug dose is the quotient of the drug dose available for the child in the mother’s milk [mcg/kg/day] and the drug dose administered to the mother [mcg/ mothers body weight] x 100. RID estimates infant drug exposure via breast milk as the percentage of the mother s dose that is absorbed by the baby. The following example shows the percentage for a woman weighing 60 kg and a drug dose of 150 mg (=150 000 mcg) [14],

Drugs with RID <10% are considered safe.

Some other important parameters include:

- t _0.5,_ the shorter the half-life the better.
- M/P ratio – mother’s milk/plasma ratio. A safe ratio is < 1. A ratio > 1 means that the transfer into milk is high. However, in the case of low plasma concentration, the dose taken by the child is low, even though the ratio may be high.
- T _max_– is the time to reach the maximum concentration (C_max_) of the drug in the mother’s plasma after drug administration. Breastfeeding should be given up during t _max._
- pKa – shows the pH value of the drug at which it is in balance between its ionized and non-ionized form. Typically, drugs transfer freely between mother’s milk and blood and they stay in a concentration balance. This mechanism serves as protection from cumulating the drug in the lactiferous alveolus. Low pKa means that there is a possibility of returning from milk to mother’s plasma according to differences in current concentrations. A drug with pKa >7.2 does not transfer back to the mother’s plasma, so there is drug sequestration in milk.
- EPB – the extent of protein binding represents the level of drug binding to mother’s plasma proteins. A high binding to mother’s plasma albumins results in weak drug transfer to mother’s milk.
- V_d_ – apparent volume of distribution is the sphere of a woman’s organism, to which the drug penetrates, other than the blood. A low value of V_d_ suggests the short presence of the drug in the organism. Together with a very short half-life it means very quick elimination of the drug from the organism.

$$RID=\frac{7.5\;mcg/kg/\text{ day }}{150000\;mcg/60\;kg/\text{ day }}\times 100=0.3\text{\%}$$

- MW – molecular weight is a strong determinant of drug transfer from mother’s plasma to milk and of

absorption from the child’s digestive tract to circulation. Drugs with low molecular weight (<200 g/mol) easily penetrate the endothelium of blood vessels and the lactiferous alveolus epithelium to mother’s milk. Large molecule drugs have difficulty in passing the lipid barrier of the cell membrane, which is bound to block the drug’s access to mother’s milk.

- BA oral – bioavailability after oral administration (digestive tract absorption). The term “poor” means low drug absorption from milk through the child’s digestive tract to blood.

Drug transfer into mother's milk increases if the drug:

1. reaches a high concentration in the mother’s blood
2. has low molecular weight
3. binds poorly to plasma proteins (high unbound drug fraction)
4. is lipophilic and easily crosses phospholipid membranes.

Parameters associated with high drug bio availability to a child:

1. high relative infant dose (RID)
2. long biological half-life
3. high mother’s milk/plasma ratio
4. high apparent volume of distribution
5. long t _max_
6. high pKa.

## The risk of administering drugs in pregnancy and lactation

During pregnancy, there is a risk associated with the occurrence of congenital disorders and malformations of the fetus. Generally, it does not line up with the risk for lactation. However, there is a possibility of these drugs having a negative effect on lactogenesis 1 in the third trimester of pregnancy. They may also influence the child’s condition after birth.

The risk of using drugs during pregnancy and lactation was categorized. For 40 years we have used the ABCDX category system to evaluate the safety of a drug for use during pregnancy [16, 17]. In 2015 the Food and Drug Administration (FDA) has started to replace this system with a more comprehensive narrative labeling system based on available data, called the Pregnancy and Lactation Labeling Rule (PLLR) [18, 19]. It is addressing the safety of drugs for women who are of reproductive age, are currently pregnant or breastfeeding. These recommendations provide detailed information for both the patients and health care providers, and they include three main categories: risk summary, clinical consideration, and data. This new categorization is currently being implemented by the FDA in stages. Since more doctors are comfortable with using the old ABCDX categories, it may take some time before they disappear. The ABCDX categories are defined as follows:

## Drugs – Pregnancy categories [16, 17]

- Well-controlled studies in pregnant women show no risk to the fetus.
- Either animal studies show risk, but human findings do not, or no well-controlled studies have been conducted in humans, but animal studies show no risk to the fetus.
- Either no well-controlled studies have been conducted in humans, or animal studies have demonstrated an adverse effect on the fetus.
- Evidence of human risk to the fetus exists, however, benefits may outweigh risks in certain situations.
- Teratogens. Controlled studies in animals or humans, or post-marketing reports, demonstrate fetal abnormalities; the risk in pregnant women clearly outweighs any possible benefit. Contraindicated in pregnancy.

## Drugs – Lactation risk categories [20]

**L1** Safest (compatible). Controlled studies in breastfeeding women have shown no negative effect on a baby. Non-absorbing drugs with low bioavailability.

**L2** Safer (probably compatible). No harmful effects have been demonstrated. The safety was defined in a limited number of studies in humans. Breastfeeding is allowed.

**L3** Moderately safe. There are no controlled studies in humans. Some studies show possible biological drug impact on the baby. A drug can be used if the benefits outweigh the risks. This category covers all new, uncharted drugs.

**L4** Possibly hazardous. Documented risk for child or lactation. These drugs are administered if they are necessary for the mother and they are impossible to substitute.

**L5** Hazardous. Studies in humans have demonstrated significant and documented risk to the child. These drugs are contraindicated in lactation.

The specific characteristics of these drugs in case of their transfer into fetal blood and to mother’s milk is presented in part 2.

## Summary

1. Drugs used in obstetric anesthesia transfer into child’s blood and to mother s milk.
2. The transfer level varies. It depends on the pharmacokinetic properties of the drug used.
3. Knowledge of pharmacokinetics of drugs used in women giving birth and in women during lactation makes it possible to minimize the drug’s effect on the breastfed newborn.
